# Advances in Diagnosis of Respiratory Diseases of Small Ruminants

**DOI:** 10.1155/2014/508304

**Published:** 2014-06-15

**Authors:** Sandip Chakraborty, Amit Kumar, Ruchi Tiwari, Anu Rahal, Yash Malik, Kuldeep Dhama, Amar Pal, Minakshi Prasad

**Affiliations:** ^1^Animal Resources Development Department, Pt. Nehru Complex, Agartala 799006, India; ^2^Department of Veterinary Microbiology, College of Veterinary Sciences and Animal Husbandry, Uttar Pradesh Pandit Deen Dayal Upadhayay Pashu Chikitsa Vigyan Vishwa Vidyalaya Evam Go-Anusandhan Sansthan (DUVASU), Mathura 281001, India; ^3^Division of Pharmacology and Toxicology, Indian Veterinary Research Institute, Izatnagar 243122, India; ^4^Division of Standardization, Indian Veterinary Research Institute, Izatnagar 243122, India; ^5^Division of Pathology, Indian Veterinary Research Institute, Izatnagar 243122, India; ^6^Division of Surgery, Indian Veterinary Research Institute, Izatnagar 243122, India; ^7^Department of Animal Biotechnology, College of Veterinary Sciences, Lala Lajpat Rai University of Veterinary and Animal Sciences (LLRUVAS), Hisar 125004, India

## Abstract

Irrespective of aetiology, infectious respiratory diseases of sheep and goats contribute to 5.6 percent of the total diseases of small ruminants. These infectious respiratory disorders are divided into two groups: the diseases of upper respiratory tract, namely, nasal myiasis and enzootic nasal tumors, and diseases of lower respiratory tract, namely, peste des petits ruminants (PPR), parainfluenza, Pasteurellosis, Ovine progressive pneumonia, mycoplasmosis, caprine arthritis encephalitis virus, caseous lymphadenitis, verminous pneumonia, and many others. Depending upon aetiology, many of them are acute and fatal in nature. Early, rapid, and specific diagnosis of such diseases holds great importance to reduce the losses. The advanced enzyme-linked immunosorbent assays (ELISAs) for the detection of antigen as well as antibodies directly from the samples and molecular diagnostic assays along with microsatellites comprehensively assist in diagnosis as well as treatment and epidemiological studies. The present review discusses the advancements made in the diagnosis of common infectious respiratory diseases of sheep and goats. It would update the knowledge and help in adapting and implementing appropriate, timely, and confirmatory diagnostic procedures. Moreover, it would assist in designing appropriate prevention protocols and devising suitable control strategies to overcome respiratory diseases and alleviate the economic losses.

## 1. Introduction

Small ruminants particularly sheep and goats contribute significantly to the economy of farmers in Mediterranean as well as African and Southeast Asian countries. These small ruminants are valuable assets because of their significant contribution to meat, milk, and wool production, and potential to replicate and grow rapidly. The great Indian leader and freedom fighter M. K. Gandhi “father of the nation” designated goats as “poor man's cow,” emphasizing the importance of small ruminants in poor countries. In India, sheep and goats play a vital role in the economy of poor, deprived, backward classes, and landless labours. To make this small ruminant based economy viable and sustainable, development of techniques for early and accurate diagnosis holds prime importance. Respiratory diseases of small ruminants are multifactorial [1] and there are multiple etiological agents responsible for the respiratory disease complex. Out of them, bacterial diseases have drawn attention due to variable clinical manifestations, severity of diseases, and reemergence of strains resistant to a number of chemotherapeutic agents [2]. However, sheep and goat suffer from numerous viral diseases, namely, foot-and-mouth disease, bluetongue disease, maedi-visna, orf, Tick-borne encephalomyelitis, peste des petits ruminants, sheep pox, and goat pox, as well as bacterial diseases, namely, blackleg, foot rot, caprine pleuropneumonia, contagious bovine pleuropneumonia, Pasteurellosis, mycoplasmosis, streptococcal infections, chlamydiosis, haemophilosis, Johne's disease, listeriosis, and fleece rot [3–10].

The respiratory diseases represent 5.6 per cent of all these diseases in small ruminants [11]. Small ruminants are especially sensitive to respiratory infections, namely, viruses, bacteria, and fungi, mostly as a result of deficient management practices that make these animals more susceptible to infectious agents. The tendency of these animals to huddle and group rearing practices further predispose small ruminants to infectious and contagious diseases [6, 9]. In both sheep and goat flocks, respiratory diseases may be encountered affecting individuals or groups, resulting in poor live weight gain and high rate of mortality [5]. This causes considerable financial losses to shepherds and goat keepers in the form of decreased meat, milk, and wool production along with reduced number of offspring. Adverse weather conditions leading to stress often contribute to onset and progression of such diseases. The condition becomes adverse when bacterial as well as viral infections are combined particularly under adverse weather conditions [1]. Moreover, under stress, immunocompromised, pregnant, lactating, and older animals easily fall prey to respiratory habitats, namely,* Streptococcus pneumoniae*,* Mannheimia haemolytica*,* Bordetella parapertussis*,* Mycoplasma* species,* Arcanobacterium pyogenes*, and* Pasteurella species* [2, 4, 7–9, 12, 13]. Such infections pose a major obstacle to the intensive rearing of sheep and goat and diseases like PPR, bluetongue, and ovine pulmonary adenomatosis (Jaagsiekte) adversely affect international trade [2, 9, 10, 13], ultimately hampering the economy.

## 2. Respiratory Diseases of Small Ruminants

Depending upon the involvement of etiological agent, the infectious respiratory diseases of small ruminants can be categorized as follows [9, 14]:

1. bacterial: Pasteurellosis, Ovine progressive pneumonia, mycoplasmosis, enzootic pneumonia, and caseous lymphadenitis,
2. viral: PPR, parainfluenza, caprine arthritis encephalitis virus, and bluetongue,
3. fungal: fungal pneumonia,
4. parasitic: nasal myiasis and verminous pneumonia,
5. others: enzootic nasal tumors and ovine pulmonary adenomatosis (Jaagsiekte).

Manytimes due to environmental stress, immunosuppression, and deficient managemental practices, secondary invaders more severely affect the diseased individuals; moreover, mixed infections with multiple aetiology are also common phenomena [5, 8, 13, 15].

These conditions involve respiratory tract as primary target and lesions remain confined to either upper or lower respiratory tract [7, 16]. Thus, these diseases can be grouped as follows [5, 8, 14, 17].

1. Diseases of upper respiratory tract, namely, nasal myiasis and enzootic nasal tumors, mainly remain confined to sinus, nostrils, and nasal cavity. Various tumors like nasal polyps (adenopapillomas), squamous cell carcinomas, adenocarcinomas, lymphosarcomas, and adenomas are common in upper respiratory tracts of sheep and goats. However, the incidence rate is very low and only sporadic cases are reported.
2. Diseases of lower respiratory tract, namely, PPR, parainfluenza, Pasteurellosis, Ovine progressive pneumonia, mycoplasmosis, caprine arthritis encephalitis virus, caseous lymphadenitis, verminous pneumonia, and many others which involve lungs and lesions, are observed in alveoli and bronchioles.

Depending upon the severity of the diseases and physical status of the infected animals, high morbidity and mortality can be recorded in animals of all age groups. These diseases alone or in combination with other associated conditions may have acute or chronic onset and are a significant cause of losses to the sheep industry [3, 10]. Thus, the respiratory diseases can also be classified on the basis of onset and duration of disease as mentioned below [3, 9, 14, 18]:

1. acute: bluetongue, PPR, Pasteurellosis, and parainfluenza,
2. chronic: mycoplasmosis, verminous pneumonia, nasal myiasis, and enzootic nasal tumors,
3. progressive: Ovine progressive pneumonia, caprine arthritis encephalitis virus, caseous lymphadenitis, and pulmonary adenomatosis.

## 3. Need of Advanced Diagnostic Approaches

The potential losses due to respiratory diseases can be minimized by sound diagnostic approach along with sound management programme [15]. Any kind of compromise with the diagnostic and management approach would severely affect the health status of the flock [19]. Early, rapid, and effective diagnosis of the respiratory diseases in small ruminants is a challenge due to limited laboratory resources in African and Southeast Asian countries where a large small ruminant population gets decimated due to respiratory disease outbreaks [15, 16]. Conventional methods of diagnosis may be available more frequently but they usually take longer to yield results, and also their specificity and sensitivity may not be up to the mark. In recent past, many advanced, rapid, sensitive, and specific serological and molecular tests have been developed. These diagnostic methods have supplanted the conventional diagnostic procedures owing to their speed, sensitivity, specificity, and applicability even without isolation of etiological agent [20, 21].

**Figure 1:**
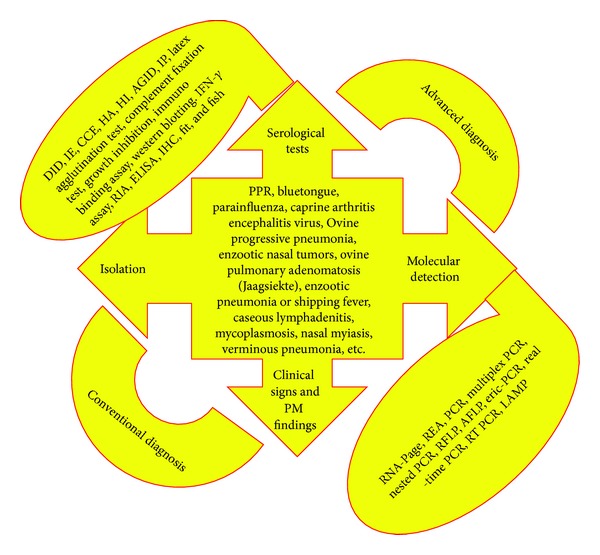
Diagnosis of infectious respiratory diseases of small ruminants.

In present scenario of globalization and regulations related to international trades, continuous monitoring of enlisted diseases is mandatory and for that sampling, isolation, and confirmation processes are very tedious [22, 23]. In such scenario, the rapid and specific detection of antibodies to the respiratory pathogens is now possible by the advancement in serological testing. Availability of better serological tests including ELISAs and monoclonal antibodies has enabled detection of antibodies to these infectious agents (namely, bacteria, viruses, and fungi) with more rapidity as well as specificity [24]. Moreover, due to advancement in the polymerase chain reaction (PCR) technology, there has been enormous improvement in the diagnosis of respiratory diseases of small ruminants [25]. Recent advances in biotechnology and molecular biology have led to the development of a variety of diagnostic assays, namely, PCR, RT-PCR, PCR-ELISA, RAPD, AFLP, RFLP, real-time PCR, quantitative PCR, multiplex PCR, LAMP, microsatellites, gene sequencing, and phylogenetic analysis, which not only help in identification but also assist in molecular characterization of various pathogens [20, 22–37]. Various conventional diagnostic tests, namely, isolation, postmortem finding, and gross clinical examinations along with modernized serological and molecular tests, are enlisted in Figure 1.

Advances in diagnostic tools and assays help strengthening the surveillance and monitoring systems of animal diseases. The latest advances in molecular techniques have assisted in the rapid and confirmatory diagnosis of the diseases and epidemiological studies to formulate appropriate and timely prevention, treatment, and control measures, and alleviation of economic losses to animal producers [1, 7, 13, 22, 23].

## 4. Advances in Diagnosis of Respiratory Diseases of Small Ruminants

For the prevention and control of fatal infectious respiratory diseases of small ruminants, various diagnostic strategies are adopted worldwide. The diagnostic tests as well as procedures adopted in different parts of world incorporate combination of conventional and advanced diagnostic tests. However, the initial suggestive diagnosis involves the observation of clinical signs and postmortem findings followed by serological and molecular methods for the confirmation of etiological agents. Common infectious respiratory diseases of small ruminants, clinical signs, postmortem findings, and diagnostic tests are compiled in Table 1.

### 4.1. Peste des Petits Ruminants (PPR)

Peste des petits ruminants (PPR) is an acute and highly contagious viral disease of small ruminants and in particular of goats, popularly known as goat plague [38, 39]. Transmission of the disease takes place by direct contact with the secretions or excretions from the infected animals to healthy ones, which are in close contact. Clinically, PPR is characterized by pyrexia, ocular and nasal discharges, erosive stomatitis, and diarrhea [38, 40]. The postmortem findings are limited mainly to the alimentary tract that consists of erosive stomatitis (extensive in nature) as well as hemorrhagic gastroenteritis. Often, streaks of congestion may be found along the mucosal folds that result in the characteristic appearance of “zebra-strip” [131, 132]. The morbidity and mortality rates of PPR can be as high as 100% and over 90%, respectively [39, 40].

The various serological tests applied in the PPR detection include agar gel immunodiffusion, virus neutralization, complement fixation, haemagglutination inhibition, and competitive ELISA assays. Conventional serological tests like complement fixation or haemagglutination inhibition cannot differentiate between PPR and Rinderpest (RP). However, haemagglutination inhibition (HI) can be used quantitatively for the measurement of PPRV antibodies in suspension. Titration of the PPRV antigen can be done by the use of both haemagglutination (HA) and HI tests [39–41]. Peste des petits ruminant's virus (PPRV) can be differentiated from Rinderpest (RP) by virus neutralization and competitive ELISA assays. Competitive ELISA can be a better choice for detection of antibody to PPR because of its high specificity of diagnosis [40]. A rapid as well as sensitive and virus-specific test for detection of PPRV antigen is immunocapture ELISA that can cause differentiation of RP and PPR. It has got higher sensitivity than routinely used agar gel immunodiffusion test [36, 42, 133].

There has been a substantial improvement in the techniques to detect the nucleic acids of PPRV. PCR assays are now considered as powerful as well as novel means of detection and quantification of the nucleic acids of PPR virus in various types of clinical samples. But unfortunately, no single assay can detect all the lineages of the virus. Companion tests can be developed by manipulation of the PPRV gene and insertion of either positive or negative markers [25, 29, 30]. The nucleoprotein based RT-PCR, which is based on nucleoprotein (N) genes, has been standardized recently. Instead of analysis of the amplified product by means of agarose gel electrophoresis, its detection is done on a plate by ELISA using labeled probe. The sensitivity of this RT-PCR ELISA is ten times higher than the classical RT-PCR. With the aid of quantitative real-time RT-PCR, there has been significant improvement in the diagnosis of PPR [29, 30]. This minimizes the risk of contamination. There is also description of applying nucleic acid amplification for the diagnosis of PPR. The sensitivity of this assay is similar to that of PCR but its simplicity in implementation, as the results can be read by naked eye, and rapidity make it suitable for practical application [32, 34].

Use of LAMP significantly reduced the processing time of sample and final outcome [21]. Similarly, LAMP assay based on conserved region of “N” gene of PPR virus has been documented for rapid and specific detection of PPR virus from clinical samples. The assay was found 100–1000 times superior to PCR and s-ELISA [43].

Synthetic peptide and multiple antigenic peptide based antigen has been employed in ELISA for detection of PPR virus antibodies. A PCR-ELISA based on N gene has been standardized in order to detect PPR virus thereby yielding a product (which is labeled with digoxigenin) that comprises a sequence from N gene of the PPRV. The assay has been found to be more sensitive than sandwich ELISA in order to detect the virus in both early and late phases of the disease. For differential diagnosis of PPRV from Rinderpest virus, also the assay has been found to be useful [27]. A one-step multiplex RT-PCR (single tube) has been standardized for amplification of specific fragments of the N as well as M genes of PPR virus. For detection of the virus directly from clinical field samples, the RT-PCR is conducted by the use of purified viral RNA. The assay is easier than the two-step assay as it is time saving requiring only one buffer for both reverse transcription and PCR [29]. RT-PCR based on F gene has shown a low sensitivity as well as specificity along with moderate agreement as compared to sandwich ELISA [36]. By the use of one-step Brilliant SYBR Green Kit, a sensitive as well as rapid single step real-time RT-PCR has been standardized for detection and semiquantitation of PPRV by the use of primers specific to viral RNA and matrix protein gene. They have been compared with conventional RT-PCR as well as Taqman RT-PCR. It has been found that the assay is more rapid as well as sensitive than TaqMan and the conventional RT-PCR in order to detect nucleic acid of PPRV from the clinical samples of sheep as well as goat, which are suspected for PPR. As an alternative test to the various diagnostic assays that already exist, SYBR Green RT-PCR has been found to be a successful tool thereby helping in rapid clinical diagnosis with advantage of reducing contamination risk [30, 37].

### 4.2. Bluetongue

Bluetongue (BT) is one of the important infectious diseases of domestic and wild ruminants. It is caused by bluetongue virus (BTV) of genus* Orbivirus* and family Reoviridae. The disease, transmitted by* Culicoides* (biting midges), was first reported in India in 1964 [134]. India has significant populations of domestic and wild ruminants, which are known to be susceptible to BTV infection. Several exotic breeds of sheep were introduced into the country between 1960 and 1970 for genetic improvement of the national flock by crossbreeding with native breeds [135]. This increase in the susceptible population, along with favorable climatic conditions, appears to have led to the establishment of BTV in the country [135, 136]. The disease has an incubation period of 5–20 days with the development of symptoms within a month. There is low mortality rate but in susceptible breeds of sheep the mortality may be high [136]. Asymptomatic infection is usually observed in cattle as well as goats and wild ruminants despite the high level of virus in the blood. Exception is red deer in which the disease may be as acute as in sheep [137]. The development in diagnostic technologies has confirmed over the past that BTV is now widely spread in several parts of India [136, 138].

Traditionally, the diagnosis of BTV is primarily based on clinical signs and symptoms. However, differential diagnosis with some of the diseases such as contagious ecthyma, foot and mouth disease (FMD), vesicular stomatitis, malignant catarrhal fever (MCF), bovine virus diarrhea (BVD), infectious bovine rhinotracheitis (IBR), parainfluenza-3 infection, and sheep pox should be done [135, 136, 139]. The confirmatory diagnosis may be done either through virus isolation or through serological test. The virus isolation is performed in embryonated chicken eggs, in cell culture (BHK-21 or Vero cell line), or occasionally in sheep [139]. The virus is serotyped either by virus neutralization tests such as plaque reduction, plaque inhibition, Microtiter neutralization, and Fluorescence inhibition test (FIT) or through reverse-transcription polymerase chain reaction (RT-PCR) (a prescribed test for international trade) [50, 51]. A highly sensitive silver staining method of RNA-polyacrylamide gel electrophoresis (RNA-PAGE) of bluetongue virus was developed recently [49]. Various serological tests such as complement fixation test (now largely replaced by the AGID test), agar gel immunodiffusion, and competitive enzyme-linked immunosorbent assay (both are prescribed test for international trade) are used for serological characterization of BTV. Recently, novel Indian isolates of BTV 21 were detected employing real-time PCR assay [44]. The complete genome sequence of BTV serotype 16 of goat origin from India has also been carried out [140]. Similarly, the complete genome sequences of BTV22 and reassortment strain of BTV 2, 3, 16, and 23 from India have been carried out recently [45–48, 52]. Analyses of the nucleotide sequence as well as phylogenetic comparisons of genome segment 2 that encodes outer-capsid protein VP2 help in creation of segment-2 database [53]. Such database is used for developing rapid as well as reliable typing assay based on RT-PCR [50, 51, 54]. Testing of multiple primer pairs has also been done that provides an identification of serotype initially by amplifying a cDNA product of the expected size. Confirmation of serotype has been done by sequencing of the cDNA amplicons and subsequently phylogenetic analysis is done for comparing with reference strains that are previously characterized [52, 54]. The RT-PCR assay provides a rapid as well as sensitive and reliable method to identify and differentiate all the serotypes of BTV [45, 50, 51, 55–58].

### 4.3. Parainfluenza

Parainfluenza is mainly characterized at necropsy by purulent bronchopneumonia (focal) along with moderate to severe pulmonary congestion. Histopathological analysis has revealed the presence of acute and severe as well as diffuse necrotizing and fibrinous or suppurative bronchopneumonia. There is also a presence of diffuse congestion as well as pulmonary edema [61]. As a diagnostic method, comparison of enzyme immunoassay has been done with complement fixation test (CFT). The cross-reactivity of the viruses can be detected by the application of such tests [59]. Parainfluenza is a viral infection of the lower respiratory tract causing an enormous burden of disease in small ruminants. Direct immunofluorescence technique along with cross-neutralization tests is required for antigenic analysis of the parainfluenza virus isolates. For detection of the virus associated with it, new diagnostic test like multiplex PCR has got enormous advantages mainly because of its specificity [17]. Real-time PCR (RT-PCR) is a useful molecular tool for detection of parainfluenza virus type 3 (Pi3) from ribonucleic acid (RNA) samples from cells of the lungs from the slaughtered animals. This is followed by sequencing as well as restriction enzyme patterns of the fragment amplified of the F gene which confers confirmation of the distinctness of the isolates. Availability of suitable PCR primers allows detection of the ovine virus specifically [62]. Phylogenetic analysis of the amino acid as well as the nucleotide sequences is also equally important [60]. In some of the instances, it has been seen that the in-house RT-PCR methods cannot yield expected products for which the nucleotide sequence analysis has been initiated [63]. Multiplex RT-PCR can help distinguish parainfluenza viruses from other respiratory virus like adenovirus [64]. Nucleic acid sequence based amplification (NASBA) has been developed for which primers as well as probes have been selected from the haemagglutinin-neuraminidase (HN) gene as well as from the phosphoprotein (P) of the parainfluenza virus [61, 65].

### 4.4. Caprine Arthritis Encephalitis Virus

Caprine arthritis encephalitis virus (CAEV) is a member of the lentivirus family (in small ruminants) leading to chronic disease of the joints and rarely encephalitis in goat kids under the age of six months. The virus is in close intimation with white blood cells. Thus, any kinds of body secretions containing blood cells are potential sources for virus spread to other animals in the herd [141, 142]. In goats,in order to detect caprine arthritis encephalitis virus (CAEV), serological tests or cell cultures are mainly used. Besides, PCR has also been developed for detection of CAEV sequences from peripheral blood mononuclear cells (PBMC), synovial fluid cells (SFC), and milk cells (MC) from the infected goats. This type of PCR assay especially provides a useful method to detect CAEV infection in goats [66–68]. A two-step TaqMan quantitative (q) PCR, which is specific as well as sensitive for the detection of infection due to CAEV by the use of a set of primers (specific), and a TaqMan probe that targets a region which is highly conserved within the gene that encodes the capsid protein of the virus have been developed [33]. In the total deoxyribonucleotide (DNA) extracts, the proviral DNA can be detected successfully by this assay. The TaqMan qPCR assay provides a fast as well as specific and sensitive means for detection of proviral DNA of the virus and thereby proves to be useful for detection in large scale for eradication programs as well as epidemiological studies.

PCR techniques have been standardizedin several laboratories for the detection of proviral DNA. Other molecular techniques such as cloning and sequencing are also used to provide knowledge on a country or region's specific strain of CAEV. Phylogenetic analyses of the proviral DNAs of CAEV throughout the world have given the suggestion that in certain areas CAEV causes natural infection not only in goats but also in sheep. In order to track the transmission of the disease in near future, phylogenetic analyses may be used [66, 69, 70]. Molecular techniques such as cloning and sequencing are also used to provide knowledge on the prevalence of specific strain of CAEV in a country or a region which may have influence on serological assay as well as corresponding CAEV antigen [33, 71].

### 4.5. Ovine Progressive Pneumonia (Maedi-Visna)

Most of the sheep suffering from Ovine progressive pneumonia (OPP) do not show the clinical signs until the age of 2 years due to the long incubation period of the virus. General loss in body condition known as the “thin ewe syndrome” is the first sign of the disease. There may be loss of weight in spite of the normal appetite of the affected sheep [143, 144]. Several serological tests like agar gel immunodiffusion (AGID), immunoprecipitation (IP), and competitive ELISA (cELISA) are used for the diagnosis of Ovine progressive pneumonia with the use of methionine-labelled antigen A [73]. Real-time quantitative PCR (qPCR) which is specific for the transmembrane region of the envelope gene (tm) has been compared with competitive inhibition enzyme-linked immunosorbent assay (cELISA) using sheep sera. The qPCR assay indicates excellent agreement between the two tests. Both disrupted whole virus and recombinant viral proteins have been utilized in indirect ELISAs which have shown high sensitivity as well as specificity of detection [73]. Such experiments have proved that the proviral loads of Ovine progressive pneumonia virus (OPPV) qPCR can be confirmed by cloning as well as sequencing and can be used as diagnostic tool for OPPV infection as well as measurement of viral load in sheep which are infected [74, 75]. Single enzyme-based automated immunohistochemical (IHC) analysis has been developed to detect capsid antigen (CA) of OPPV that uses two anti-CAEV monoclonal antibodies, namely, 5A1 as well as 10A1 along with two enzyme-based IHC systems. The CA of OPPV has been detected in the intracellular regions of the synovial membrane of the carpus, in the cells that resemble alveolar macrophages as well as interstitial macrophages in the lung tissue, and so also in alveolar cells of the mammary gland [76]. Comparison of a new real-time quantitative PCR (qPCR) which is specific for the envelope gene's transmembrane region has been done with a competitive ELISA (cELISA). Such comparative test has led to the conclusion that qPCR may be used as a supplemental tool for diagnosis and for measuring the load of the virus [71, 145].

### 4.6. Enzootic Nasal Tumors and Ovine Pulmonary Adenomatosis (Jaagsiekte)

From the diagnostic point of view of enzootic nasal tumors and ovine pulmonary adenomatosis, it is important to note that the genome of the ovine pulmonary adenomatosis virus is 7,434 nucleotides long thereby exhibiting a genetic organization of type B as well as D oncoviruses. The enzootic nasal tumor virus is closely related to the Jaagsiekte retrovirus of sheep as well as to sheep endogenous retroviruses [146, 147]. Diagnosis of enzootic nasal tumors is based on mainly clinical findings. Endoscopy reveals occlusion in the caudal part of one or both the nasal cavities. Radiography may also reveal the extent of the lesion. Provisional diagnosis can be made by the biopsy of the mass during the period of endoscopic examination [80]. RT-PCR for the diagnosis of Jaagsiekte is very important in order to formulate prevention as well as control strategies. The envelope (env) gene is mainly targeted for this purpose [81]. For development of an assay based on serology, identification of three proteins has been done as candidate diagnostic antigens, namely, Jaagsiekte sheep retrovirus (JSRV) p26 (which is a group specific antigen), the transmembrane, and the open reading frame (ORF)-X proteins. Isolation of the genes coding for all the three proteins has been done followed by cloning as well as expression. Purification of the JSRV p26 has been done as a potential diagnostic antigen by both Western blot and ELISA. Investigation of three molecular assays has been done for their sensitivity as well as specificity: the long terminal repeat (LTR) group specific antigen (gag) PCR, LTR heminested PCR, and the PCR covering the V1 or V2 region. The use of AmpliTaq gold DNA polymerase increases the specificity of heminested PCR. The complete genome sequence of the ovine enzootic nasal tumor virus has been done which has shown its exclusive association with contagious intranasal tumors of sheep [79, 82, 83].

### 4.7. Enzootic Pneumonia or Shipping Fever

Before discussing enzootic pneumonia in sheep, it has to be kept in mind that as far as the transmission of the disease from diseased to healthy animals is concerned, no direct evidence is available yet. As per suggestion, it has been noted that there may be precipitation of outbreaks due to abrupt environmental changes and it may also be associated with a sharp change in weather conditions [86, 87]. Such infection in animals caused by a bacterial species related to genus* Pasteurella* is known as Pasteurellosis. After the taxonomic revision in 1999, the species is classified as* Mannheimia species*.* Pasteurella multocida (P. septica)* is carried in mouth and respiratory tract of several animals, notably cats. The organisms are small Gram-negative bacillus with bipolar staining.* P. multocida*, a common commensal, causes numerous pathological conditions in domestic animals, avian species, and human beings. Pasteurellosis is associated with a close animal contact and may be transmitted by animal bite [88, 89]. Severe clinical conditions occur when the organism is associated with other infectious agents, such as mycoplasma, chlamydia, and viruses [7, 9]. Environmental conditions and various stress factors such as transportation, housing deficiency, and bad weather also play a role to further aggravate the clinical conditions. Among the various diseases considered to be caused by* P. multocida*, alone or in association with other pathogens, most important is shipping fever in cattle and sheep, which may also be caused by* Mannheimia haemolytica*, in the absence of* P. multocida*. Fresh samples are the prerequisites for isolation of* Pasteurella multocida* and subsequently demonstration of the bipolar staining characteristic. A wide range of media that can be used for isolation of the organism are blood and chocolate agar and casein/sucrose/yeast (CSY) agar with supplementation of 5% blood. Other media include dextrose starch agar as well as trypticase soy agar. For demonstration of the characteristic staining feature, methylene blue or Leishman's stain is usually used. For serotyping, the tests include rapid slide agglutination test as well as indirect haemagglutination test (for capsular typing); for somatic typing an agglutination test; and agar gel immunodiffusion for both capsular and somatic typing. For the rapid identification of capsular type, counterimmunoelectrophoresis is an important diagnostic tool. Dot immunobinding assay, immunoblotting of outer membrane proteins of vaccine, and field isolates of* Pasteurella multocida* have been used for rapid diagnosis [90, 91]. Comparative analysis of the outer membrane protein profiles of haemorrhagic septicaemia associated* P. multocida* by immunoblotting studies indicated that the major OMP of* P. multocida* (B: 2) is highly antigenic and 37 kDa OMP has potential for protective and immunodiagnostic studies [92].

In clinical samples as well as bacterial cultures, detection of organisms can be done by PCR. The pair of primers for this particular assay can amplify a 353 base pair (bp) fragment of the 16srRNA gene, which ultimately results in the amplification of DNA. Thus, this kind of PCR assay usually represents a valuable tool for diagnosing the disease early ultimately facilitating better control of the disease. Similar strategies can be adopted for the identification and confirmation of enzootic pneumonia in sheep with advanced molecular methods [20, 35].

For epidemiological investigations, characterization of isolates can be done by DNA fingerprinting but availability of such diagnostic test is restricted to research laboratories [85, 93]. Southern hybridization can lead to confirmation of the presence of the bacterial sequence, which is often suggestive of the virulence of the organism [94]. Upon presumptive or definitive diagnosis, further differentiation of isolates can be achieved by genotypic fingerprinting methods. Restriction endonuclease analysis for characterization of serotypes of hemorrhagic septicaemia can be done with the enzyme HhaI. Discrimination of the isolates can be done by application of ribotyping as well as large DNA separation by means of pulsed-field gel electrophoresis. The rapidity as well as reproducibility of AFLP is high with higher index of discrimination. PCR fingerprinting is feasible in any laboratory, which has got the PCR capability. RAPD analysis as well as arbitrarily primed PCR (AP-PCR) is found to be useful for epidemiological investigation. For discriminating sheep as well as goat isolates, repetitive sequence PCR is also found to be useful. Repetitive extragenic palindromic REP-PCR as well as single prime PCR has been found to be useful for differentiating various serogroups of the bacteria [95, 96].

### 4.8. Caseous Lymphadenitis

The disease is caused by* Corynebacterium pseudotuberculosis*. There are two basic forms of caseous lymphadenitis, that is, internal form and external form. Most of the affected animals manifest both forms of the disease depending on the multiple factors that are age, physiological conditions, environmental factors, and managemental practices [148]. There is obvious nodule formation under the skin as well as enlargement of peripheral lymph nodes in the external form. The affected lymph nodes along with the subcutaneous tissues are enlarged with thick as well as cheesy pus which may rupture outward spontaneously or during the process of shearing or dipping. The internal form of caseous lymphadenitis (CLA) is manifested by vague signs such as weight loss, poor productivity, and decrease in fertility [3, 148, 149]. For the detection of the causative agent,* Corynebacterium pseudotuberculosis*, in sheep and goats, a double antibody sandwich ELISA has been developed, which has been further modified for improving the sensitivity. The main objective of developing this test is to detect the presence of antibodies against the bacterial exotoxin. It has been found that six proteins with varying molecular mass ranging from 29 to 68 kilo Dalton (kDa) react with sera from both goats and sheep acquiring infection experimentally or naturally. For classification of the sera with inconclusive results, immunoblot analysis has been found to be valuable [100, 101]. Quantification of interferon gamma (IFN-*γ*) is essential for accurate diagnosis of the disease for which an ovine IFN-*γ* ELISA has been developed. The sensitivity of the assay is slightly more for sheep than in goats while the specificity of the assay is higher for goats than for sheep. It can thus be concluded that IFN-*γ* is a potential marker in order to determine the status of CLA infection in small ruminants [102]. For the diagnosis of CLA, another novel strategy is the employment of PCR for identification of the bacteria isolated from abscesses [103]. The PCR has been found to be both sensitive and specific in addition to its rapidity of detecting* C. pseudotuberculosis* from sheep that are naturally infected [99].

### 4.9. Mycoplasmosis

As far as the antigenic variation is concerned, mycoplasmas have complex mechanisms enabling them to evade the immune system. They thereby cause several clinical symptoms which are having significant economic effect on production of small ruminants [107]. There are many species in genus* Mycoplasma* associated with pneumonic and respiratory conditions in small ruminants, namely,* Mycoplasma agalactiae*,* Mycoplasma mycoides subspecies mycoides*,* Mycoplasma bovis*,* Mycoplasma capri*,* Mycoplasma capripneumoniae*,* Mycoplasma capricolum*,* Mycoplasma putrefaciens*, and many others [7, 9, 104, 106–108]. Mycoplasmainfection associated syndromes range from septicemia (acute) along with death to chronicity of infection that results in reduced production [150]. Pneumonia accompanied by mastitis, keratoconjunctivitis, abortions, and arthritis is commonly observed in mycoplasma syndrome [7, 9, 151]. The conventional methods for diagnosis of mycoplasmosis include isolation of caprine and ovine mycoplasma in modified Hank's Balanced Salt Solution Liquid Media (MBHS-L), followed by biochemical characterization and staining [7, 9, 105]. Initially, serological tests like growth inhibition, agar gel immunodiffusion, counter current electrophoresis, complement fixation, PAGE, and others were performed [110]. However, cross-reactivity of closely related species could not be differentiated by these serological tests [7–9, 104]. Immunobinding assay with polyclonal sera was able to differentiate closely related species [111]. It was followed by preparation of different antigens and purification with PAGE and SDS-PAGE in an attempt to identify potent specific immunogenic proteins of diagnostic values [112, 113]. Moreover, detection of protective and cross-reactive proteins with SDS-PAGE and immunoblotting showed some glimpse of diagnostic value [9, 114, 115, 151]. These proteins provided base for selective and specific tests. Development of monoclonal antibodies based on such purified and specific immunogenic proteins led to development of very sensitive and specific sandwich ELISA based on monoclonal antibodies [116]. Molecular detection of* Mycoplasma* species based on different set of primers was used to identify different species [26]. For the development of monoclonal antibody based serological as well as ELISA-PCR, identification of species specific non-cross-reactive immunogenic proteins is mandatory, and for that proteins separated in SDS-PAGE were subjected to western blotting with homo- and heterologous sera against* Mycoplasma agalactiae* and* Mycoplasma bovis* [9, 114, 115, 151]. These species specific immunogenic proteins can form the basis for development of many advanced diagnostic procedures for the detection of mycoplasma and its species confirmation.

Nowadays, for the molecular diagnosis of several clusters as well as groups, species specific primers along with restriction enzymes are used for confirmation of the agent by PCR as well as PCR-RFLP [107]. Still the combination of conventional and recently developed molecular methods is recommended for the identification and confirmation of contagious caprine pleuropneumonia (CCPP) in field outbreak [117]. For this purpose, growth inhibition test has been employed for identification of the agent followed by PCR. These two tests in particular detect two species of* Mycoplasma*, namely,* Mycoplasma capricolum* and* Mycoplasma putrefaciens* from nasal swab and lung cultures [118]. A multiplex real-time PCR has been developed for differentiation of the various* Mycoplasma* species of sheep and goat including* Mycoplasma agalactiae*. This assay particularly targets the two specific housekeeping genes, namely, polC and fusA considering which specific diagnostic primers and probes are to be developed [105, 106]. It is however important to note that the assay requires further assessment of clinical specimens but for diagnosis on large scale basis the assay is very promising [119]. Primers specific to* Mycoplasma conjunctivae* (that causes pink eye in sheep and goat) have been used for amplification of a 750-base-pair fragment of the genome through PCR, which has been subsequently confirmed by agarose gel electrophoresis [107, 109].

### 4.10. Nasal Myiasis

Both double immunodiffusion (DD) and indirect haemagglutination (IH) tests are used for detection of the somatic crude antigen first (L1) as well as second (L2) and third (L3) in star of the larva of the parasite* Oestrus ovis* [122]. For postmortem examination, sagittal sectioning of the head of the sheep suspected of suffering from nasal myiasis is carried out for detecting the presence of maggots or larvae [152]. It has been observed that there is no development of cross-immune reaction in sheep, which are naturally parasitized with all the three larval stages (as detected by DD test) and with L2 larvae (as detected by IH test) [124]. It is important to note that rhinoscopy examination can confirm the diagnosis and is equally important in treating the patient by removing the maggots with forceps [123]. For detection of seropositivity, ELISA is employed using a crude L2 larva as antigen [121]. Development of a direct ELISA by the use of a crude somatic antigen was developed from the first stage larva (L1). Validation of such system has been done with sera from both endemic and nonendemic areas [125]. The sensitivity as well as the specificity of the assay has been found to be high by the use of a cut-off point. PCR as well as automated sequencing technologies have been developed for molecular diagnosis of the disease [128]. PCR-RFLP has been used widely for identifying taxa of the parasite which are closely related and have forensic relevance [120]. It is also important to note that a better understanding of several target genes like mitochondrial DNA (mt DNA) as well as ribosomal DNA (rDNA) is pertinent for understanding the evolution of the parasite and so also for characterization of the proteins of the parasites [120, 123].

### 4.11. Verminous Pneumonia

In goats* Muellerius capillaris* is the most common lung worm. There is diffused pneumonia in affected goats without the presence of any nodular lesion. The parasite predisposes animals to secondary infections thereby compromising with the health in general [129]. A rapid as well as inexpensive method for assessment of herd exposure to lung worm in cattle is the bulk milk ELISA. It is a useful tool for the veterinary practitioners as a herd health monitoring programme component or in the perspective of investigation of herd health [126]. Over the past 15 years, studies have been conducted to prove that sequences of the internal transcribed spacers of ribosomal DNA provide useful genetic markers. This makes the basis for the molecular diagnosis of parasitic pneumonia in sheep and goat using PCR [130]. DNA probes as well as assays based on PCR are used for identification and detection of* Dictyocaulus* as well as* Protostrongylus*. The sensitivity of most of the PCR-based assays is more than DNA probe assays. Multiple steps are required for the development of assays based on PCR, which follows the selection of oligonucleotide primers at the initial stage along with reporter probe. It has been found that usually PCR detects the parasitic DNA but certainly advances have been made in preparing samples. For this purpose, it is required to extract the DNA while removing the PCR inhibitors. This helps in achieving greater sensitivity [128].

## 5. Other Unusual Complications of Respiratory Tracts

The respiratory diseases of small ruminants are generally fatal to lambs and kids. The lamb and kid pneumonia are mostly regarded as a complex of disease. It involves interaction of host related factors (immunological and physiological) and etiological agents, namely, virus, bacteria, mycoplasma, and environmental factors [4, 7, 108]. Many times, immunosuppression, malnutrition, and adverse climatic conditions lead to infection due to unusual infectious agents. There are reports on* Streptococcus pneumoniae*, commensal bacteria of the nasopharynx of animals associated with a majority of cases of morbidity and mortality in young lambs due to pneumonia [7, 13, 153]. Similarly, many other unusual pathogens* Haemophilus ovis* [154],* Streptococcus* spp.,* Pasteurella* spp. [5, 6],* M. bovis* in sheep [7, 12] and goat [155],* Mycoplasma arginini* [12], and* Haemophilus somnus* [156] may cause pneumonia. Many time mixed infections are observed. Thus, isolation and identification of such samples are always tedious to perform [7, 9]. The use of monoclonal antibodies based serological tests has simplified the process of early and specific diagnosis of many of these pathogens [24]. Simultaneously, development of molecular techniques like PCR particularly multiplex PCR is very useful for the identification and differentiation of etiological agents from such complex conditions [21].

## 6. Conclusion and Future Perspectives

For effective control of respiratory diseases of sheep and goat, accurate diagnosis along with genetic characterization of the causative agents is essential. It is especially important in present context of increased antibiotic and anthelmintic resistance. The known limitations of the traditional diagnostic techniques have created urgency to give a boost to the development of molecular diagnostic techniques along with establishment of traditional serodiagnostic facilities for overall progress in the field of respiratory disease diagnosis. Advancement in development and standardization of various kinds of PCR techniques along with genetic characterization of the causative agent has provided a solid foundation to develop practical as well as highly sensitive and specific diagnostic tools to help conduct epidemiological investigation and devise control programmes. Multiplexing of PCR assays has decreased confusion of diagnosticians in case of mixed infection as such assays can certainly differentiate between the various species within the same genera of pathogenic organism. It is obvious that advancement in the field of bionomics and biotechnology has led to the rapid and accurate diagnosis of many of these economically important diseases. All such efforts ultimately will lead to improvement of economic status of stakeholders of small ruminant husbandry and their sustainability. Development in field of nanotechnology has led to evolution of nanomedicine with the aid of which it will be certainly possible in near future to make further progress in the diagnosis and management of the respiratory diseases of small ruminants including wild life.

## Figures and Tables

**Table 1:** Common infectious respiratory diseases of small ruminants.

Sl. no.	Name of condition	Etiological agents	Affected species	Clinical signs	PM findings	Diagnostic tests	References
1	Peste des petits ruminants (PPR)	*Morbillivirus* (family Paramyxoviridae)	Goats and sheep	Mucopurulent nasal and ocular discharges, necrotising and erosive stomatitis, enteritis, and pneumonia	Congestion of mucosa of respiratory tract, exudates in tract, hardening of lungs mainly in anterior lobes, congestion hemorrhages, and erosion in intestinal mucosa	HA, HI, ELISA, PCR-ELISA, RT-PCR, real-time PCR LAMP	[29, 30, 37–43]

2	Bluetongue	*Orbivirus* (family Reoviridae)	Goats and sheep	Swelling of the lips and tongue and typical blue coloration of tongue, though this sign is confined to a minority of the animals. Nasal symptoms may be prominent, with nasal discharge and stertorous respiration	In sheep-oedematous face and ears, with dry, crusty exudate on the nostrils; hyperemic coronary bands of hooves; petechial or ecchymotic hemorrhages may be present and extend down the horn. Petechiae, ulcers, and erosions in the oral cavity, particularly on the tongue and dental pad. The oral mucous membranes may be necrotic or cyanotic. The nasal mucosa and pharynx may be edematous or cyanotic, and the trachea hyperemic and congested. Froth is sometimes seen in the trachea	AGID, RNA-PAGE, FIT, RT-PCR, ELISA, and real-time PCR	[44–58]

3	Parainfluenza	Paramyxovirus	Sheep and goats	Mostly associated with enzootic pneumonia	Generally followed by bacterial infection mainly secondary *Mannheimia* infection	Direct immunofluorescence, ELISA, RT-PCR, real-time PCR, and multiplex PCR	[11, 18, 59–65]

4	Caprine arthritis encephalitis virus	Retrovirus	Neurologic disease in kids and arthritis in adults	Lymphocytic mastitis and hard firm udders along with chronic wasting	Signs of secondary bacterial infection: thickening of interalveolar septa and lymphoid hyperplasia, chronic interstitial pneumonia	TaqMan quantitative PCR (qPCR)	[18, 66–71]

5	Ovine progressive pneumonia (maedi-visna)	Oncogenic retrovirus of subfamily Lentiviridae	Sheep	Chronic emaciation, weakness, dyspnea, lymphoproliferative pneumonia, meningeal arteritis with encephalitis, nonsuppurative arthritis, and lymphocytic mastitis	Characteristic firm lungs with grayish to brown discoloration, thickening of interalveolar septa, and lymphoid hyperplasia	Immunohistochemical (IHC), cELISA, AGID, IP, and real-time quantitative PCR (qPCR)	[18, 72–76]

6	Enzootic nasal tumors, ovine pulmonary adenomatosis (Jaagsiekte)	Retrovirus	Sheep and goats	Signs of inspiratory dyspnea along with seromucoid nasal discharge	Presence of uni- or bilateral tumor growth; firm, hard, grey colored lungs; lungs sink in water, and bronchi are found filled with white frothy fluid	ELISA, RT-PCR, real-time PCR, IHC, and nested PCR	[77–83]

7	Enzootic pneumonia (Pasteurellosis, shipping fever, and hemorrhagic septicemia)	*M. haemolytica*and* Bibersteinia trehalosi (Pasteurella trehalosi) *	Sheep	Dyspnea, pyrexia, dullness, depression, mucopurulent nasal discharge, oculonasal blood, and tinged discharge	Serofibrinous fluid in lungs with fibrinous adhesions leading to consolidation of lungs	Counterimmunoelectrophoresis, ELISA, PCR, multiplex PCR, IHC, ISH, AFLP, AP-PCR, DNA fingerprinting, and Southern blot	[14, 84–96]
8		*M. haemolytica* and *P. multocida *	Goats				

9	Caseous lymphadenitis	*Corynebacterium pseudotuberculosis *	Sheep and goats	Chronic emaciation, dyspnea, exercise intolerance, dullness, forced full coughing, and weight loss	Enlargement of lymph nodes with greenish colored pus	Haemagglutination test, Counterimmunoelectrophoresis, IFN-*γ* ELISA, and PCR	[14, 97–103]

10	Mycoplasma	*M. ovipneumoniae*, *M. capricolum*, *M. mycoides* subsp. *mycoides*, and *M. agalactiae *	Sheep and goats, kids may develop encephalitis	Anorexia, pyrexia, painful breathing, coughing, and sneezing	Peribronchiolar lymphocytic infiltrations are observed with diffused nonsuppurative pleuritis	Immunoblotting, immunobinding assay, growth inhibition, PCR-RFLP, and multiplex real-time PCR	[7, 18, 104–119]

11	Nasal myiasis	*Oestrusovis *	Sheep	Stamping of feet, efforts to hide nose, difficult breathing, and heavy sound in respiration	Swollen nasal membranes, plugged nostrils, and upper respiratory tract occluded with serofibrinous discharge	Double immunodiffusion (DD), indirect haemagglutination (IH) tests, ELISA, and PCR PCR-RFLP	[120–125]

12	Verminous pneumonia	*Dictyocaulus filaria*, *Protostrongylus rufescens*, and *Muellerius capillaris *	Young animals in the age group of 2–18 months	Pyrexia, coughing, rapid and painful breathing, nasal discharge, and emaciation with retarded growth	Presence of parasites and caseous exudates in lungs	ELISA and PCR	[14, 126–130]
